# The development of a lateral flow immunochromatographic test strip for measurement of specific IgA and IgG antibodies level against porcine epidemic diarrhea virus in pig milk

**DOI:** 10.1080/01652176.2024.2429472

**Published:** 2024-11-21

**Authors:** Patumporn Jermsutjarit, Dhithya Venkateswaran, Nitaya Indrawattana, Jessada Na Plord, Angkana Tantituvanont, Dachrit Nilubol

**Affiliations:** a Swine Viral Evolution and Vaccine Development Research Unit, Department of Veterinary Microbiology, Faculty of Veterinary Science, Chulalongkorn University, Bangkok, Thailand; b Biomedical Research Incubator Unit, Department of Research, Siriraj Center of Research Excellence in Allergy and Immunology, Faculty of Medicine Siriraj Hospital, Mahidol University, Bangkok, Thailand; c Affinome Co., Ltd., Bangkok, Thailand; d Department of Pharmaceutics and Industrial Pharmacy, Faculty of Pharmaceutical Sciences, Chulalongkorn University, Bangkok, Thailand

**Keywords:** Porcine epidemic diarrhea virus, rapid immunochromatographic test strip, IgA, IgG, milk

## Abstract

Porcine epidemic diarrhea virus (PEDV) causes severe enteric disease and high mortality in neonatal piglets, leading to significant economic losses in the swine industry. Considering that passive lactogenic immunity is crucial for preventing infection in piglets, necessitating a rapid and accurate tool to measure immunity levels. This study aims to develop a lateral flow immunochromatographic strip (LFICS) to assess IgA and IgG antibodies in colostrum and milk, using PEDV S protein. The performance of LFICS was compared to viral neutralization (VN) and enzyme-linked immunosorbent assay (ELISA) as reference methods, with a visual scoring system applied for field monitoring. Colostrum (*n* = 82) and milk (*n* = 106) samples were analyzed, showing strong correlation with reference methods and no cross-reactivity with other pig pathogens. The LFICS exhibited high relative sensitivity (Se) and specificity (Sp), with colostrum showing 98.73% Se and 66.67% Sp for IgA, and 96.15% Se and 75.00% Sp for IgG. Milk demonstrated 95.60% Se and 80.00% Sp for IgA, and 84.88% Se and 85.00% Sp for IgG. These findings indicate that the LFICS is a reliable, simple, and rapid method for measuring PEDV-specific IgA and IgG levels, offering valuable support for monitoring herd immunity and evaluating vaccination programs.

## 1. Introduction

Porcine epidemic diarrhea virus (PEDV), an enteric disease in pigs, belongs to genus *Alphacoronavirus* within the family *Coronaviridae*, which is part of the order *Nidovirales* (Schoch et al. 2020). PEDV was first identified in UK and Belgium during 1977-1978 (Wood 1977; Pensaert and de Bouck 1978). Since its discovery, the virus has caused outbreaks in numerous Asian countries, including South Korea, Japan, China, Vietnam and Thailand. These outbreaks have resulted in significant economic challenges for the global swine industry (Li et al. 2012; Song and Park 2012; Temeeyasen et al. 2014; Vui et al. 2015; Cheun-Arom et al. 2016).

PEDV primarily targets the gastrointestinal (GI) tract of pigs, specifically the intestinal villous enterocytes in the small intestine, which are the primary cells infected by the virus (Saeng-Chuto et al. 2021; Jermsutjarit et al. 2024). Villous atrophy and degeneration of enterocytes on the mucosal surface results in watery diarrhea, dehydration, and metabolic imbalance, which are reflected in clinical signs and can lead to death (Stevenson et al. 2013). The PEDV affects pigs at all ages, with high mortality, approaching 100% in neonatal piglets less than a week of age (Wood 1977; Jung et al. 2020). Barrier integrity encompasses multiple mechanisms, including tight junctions at the mucosal surface that protect against pathogens and toxins, and stimulate innate and adaptive immune responses when infections occur (Blikslager et al. 2007). Despite these protective mechanisms, barrier integrity is notably compromised in nursery piglets and undergoes significant alteration in weaned pigs (Smith et al. 2010). Hence, lactogenic immunity is critically important for neonatal piglets, as it plays a crucial role in protecting them from PEDV infection.

Lactogenic immunity is passive immunity transferred to neonatal piglets *via* colostrum and milk. This immunity primarily involves neutralizing antibodies (nAbs), which play a critical role in blocking PEDV infection at intestinal villous enterocytes (Gong et al. 2018). The nAbs can prevent PEDV infection by neutralizing virus particles, obstructing the virus’s entry into cells, and inhibiting the uncoating of the PEDV genome (Klasse and Sattentau 2002; Gong et al. 2018). Additionally, sow mammary secretions contain other crucial components, including immunoglobulins (Ig), immune cells, growth factors, and nucleotides, all of which contribute to the overall antibody response and the vitality of piglets (Schlimme et al. 2000; Blum and Baumrucker 2002). Three main types of Ig in colostrum and milk are IgA, IgG, and IgM. (Bourne and Curtis 1973). IgG is the predominant Ig in colostrum. It is absorbed through the piglet’s intestines and provides systemic antibodies to protect systemic system (Langel et al. 2016; Poonsuk et al. 2016). IgG levels drop significantly for 5 times within 24 h, while IgA becomes the dominant type in milk 2-3 days after post-parturition, providing local immunity against intestinal infections in piglets (Curtis and Bourne 1971; Foisnet et al. 2010). IgM has the smallest concentration in colostrum and milk. It responds in the serum during the early phase of PEDV infection, but there is limited available data on its function in pig milk (Guo et al. 2020). Lastly, antibody tests may help track the effectiveness of lactogenic immunity transferred from sows to their piglets in fighting PEDV infection.

PEDV, an enveloped positive-sense RNA virus, has a genome that is approximately 28 kb in length. The PEDV genome consists of two replicase polyproteins encoded by open reading frames (ORFs) 1a and 1b, followed by four different structural proteins: spike (S), envelop (E), membrane (M) and nucleocapsid (N), and one accessory protein, ORF3 (Kocherhans et al. 2001; Huang et al. 2013). The S protein, a class I viral fusion protein (Bosch et al. 2003) that is divided into 2 subunits: S1 and S2. The S1 subunit attaches to host cell receptor, while the S2 subunit facilitates membrane fusion, allowing the virus to enter host cells (Li 2016; Li et al. 2017). The S protein is crucial for stimulating the production of neutralizing antibodies in the host, making it extensively used in the development of vaccines and diagnostic reagents (Song and Park 2012). Additionally, variation in the S gene can be utilized to classify PEDV into two genotypes: classical (G1) and pandemic (G2) variants. Furthermore, the Thai PEDV variant has been subdivided into six subgroups (TH1-6) (Stott et al. 2017).

Several methods have been developed for detecting antibodies against PEDV. The viral neutralization (VN) assay is a common method for detecting neutralizing antibodies against PEDV, which block viral infectivity in cell (Paudel et al. 2014). However, this method is time-consuming, requires many instruments, and demands skilled techniques for cell culture and interpreting cytopathic effects (CPEs). Additionally, VN does not distinguish between different types of Ig in samples. Therefore, enzyme-linked immunosorbent assays (ELISAs) were developed for the evaluation of antibodies instead, but they require numerous laboratory instruments, chemical reagents, and expertise to perform and calculate antibody levels in samples, similar to VN assay. Moreover, VN and ELISA do not measure antibody values under field conditions. For a simple and rapid evaluation of antibodies, we develop a lateral flow immunochromatographic strip (LFICS) using colloidal gold-labeled S protein to detect anti-PEDV-specific IgA and IgG in pig colostrum and milk. The protective antibodies were assessed by comparison to the VN assay. The performance of the test strip was also compared to an indirect ELISA based on the S protein. Furthermore, this tool could prove beneficial for on-site evaluation of anti-PEDV measurements through semiquantitative scores and for planning preventative vaccine programs.

## 2. Materials and methods

### 2.1. Recombinant protein production, purification and detection of S protein

The S region contains neutralizing epitope (primarily in S1 and partly in S2 subunit, amino acid (aa) 210-830) was produced using the Bac-to-Bac^®^ TOPO^®^ Expression System (Invitrogen, Carlsbad, CA, USA). The Tobacco Etch virus (TEV) and the S nucleotide (nt) sequence were then incorporated into the pFastBacTM TOPO^®^ vector (Thermo Fisher Scientific, Waltham, MA, USA) using specific primers (forward primer: 5′- CTGTCCAGAGAGCTCCAGAG-3′’, reverse primer: 5′- CACGTCCAGGGTCATGAAGCT-3′’) with a 6X histidine tag at the C-terminal end, resulting in the construction of recombinant-S. Subsequently, the recombinant-S pFastBac^TM^ TOPO^®^ vector (Thermo Fisher Scientific, Waltham, MA, USA) was transformed into DH10Bac^TM^ competent *Escherichia coli* (*E. coli*) (Invitrogen, Carlsbad, CA, USA) to facilitate the transposition of the recombinant-S gene into the bacmid vector (Thermo Fisher Scientific, Waltham, MA, USA). To produce the recombinant baculovirus, the recombinant-S bacmid vector was transfected into Sf9 cells using Cellfectin^®^ II reagent (Invitrogen, Carlsbad, CA, USA) and incubated at 27 °C for 72 h until signs of viral infection were observed, resulting in the release of the baculovirus P1 designated as recombinant-S baculovirus. The P1 viral stock underwent a viral plaque assay to determine the virus titer. The recombinant-S protein was expressed by infecting the baculoviral stock into Sf9 cells at a multiplicity of infection (MOI) of 2 and incubating at 27 °C for 48 h. After incubation, the culture was centrifuged at 500 × g for 30 min to collect the supernatant for purification. The supernatant was passed through a Ni Sepharose High Performance (HP) column (HisTrap^TM^ HP; Cytiva, Marlborough, MA, USA), and the purified S protein was desalted using an Amicon Ultra-15 centrifugal filter with a 50 kDa cutoff (Millipore, Temecula, CA, USA), and finally stored at −80 °C.

The S rotein was confirmed using Native polyacrylamide gel electrophoresis (PAGE). Purified S protein was mixed with a two-fold Native sample buffer (Thermo Fisher Scientific, Waltham, MA, USA) without denaturing the protein. The mixture and NativeMARK^TM^ Unstained Protein Standard (Thermo Fisher Scientific, Waltham, MA, USA) were run through 8% Bis-Tris polyacrylamide gel at 100 V for 120 min at 4 °C. The bands of proteins were visualized by staining the gel with Coomassie Brilliant Blue (CBB). In immunoblotting analysis, the S protein separated by Native-PAGE was transferred to polyvinylidene difluoride (PVDF) and further blocked with 5% (w/v) skimmed milk powder (Merck, Darmstadt, Germany) in 0.5% Tween (Sigma-Aldrich, Burlington, MA, USA) in 1X PBS (PBS-T) for 2 h at room temperature (RT) with agitation. Blotting was performed with a 1:1,000 dilution of mouse anti-6xHis-tagged monoclonal antibody in 0.5% PBS-T (Abcam, Cambridge, UK) overnight at 4 °C with agitation. The membrane was washed 3 times with 0.5% PBS-T and then incubated with a 1:5,000 dilution of goat anti-mouse conjugated HRP in 0.5% PBS-T (Abcam, Cambridge, UK) for 2 h at RT with agitation. The membrane was washed 3 times with 0.5% PBS-T. Protein signals were detected colorimetrically using 3-amino-9-ethylcarbazole (AEC; Sigma-Aldrich, Burlington, MA, USA) substrate. The CBB stained gel and blotted PVDF membrane were captured using the iBrightTM CL1500 Imaging system (Thermo Fisher Scientific, Waltham, MA, USA).

### 2.2. ELISA analysis of S protein antigenicity using positive PEDV sera

Forty positive and negative sera from a previous study (Srijangwad et al. 2017) were used to determine the antigenic reactivity to S protein. The sera from both the exposed PEDV sows and the negative control group were confirmed using a viral neutralization (VN) assay. For the indirect ELISA, a 96-well immuno plate (Nunc, Thermo Fisher Scientific, Waltham, MA, USA) was coated with 5 µg/mL of S protein diluted in bicarbonate buffer (pH 9.6). The coated plate was incubated overnight at 4 °C. After discarding the S protein solution, the plate was blocked with 5% (w/v) bovine serum albumin (BSA, Merck, Darmstadt, Germany) solution diluted in 0.05% PBST (1X PBS, pH 7.4 containing 0.05% Tween-20) for 3 h at 37 °C.

Following the blocking step, the blocking reagent was discarded, and 1:200 diluted serum samples in 0.05% PBST containing 10% (v/v) goat serum were added to each well and incubated at 25 °C for 1 h. The plate was then washed with 0.05% PBST, and 100 µL of horseradish peroxidase (HRP)-conjugated goat anti-pig IgA and IgG (1:10,000) (Abcam, Cambridge, UK) was added, followed by incubation at 25 °C for 1 h. After discarding the secondary antibodies and washing, 3,3′,5,5′-tetramethylbenzidine (TMB) substrate (Thermo Fisher Scientific, Waltham, MA, USA) was added and incubated in the dark for 5 to 15 min at 37 °C. The reaction was stopped by adding 50 µL of 1 normal sulfuric acid (H_2_SO_4_). The optical density (OD) was measured using an ELISA plate reader at 450 nm (Metertech, Taipei, Taiwan). IgA and IgG levels were analyzed using the sample-to-positive (S/P) ratio.

### 2.3. Conjugation of S protein and mAb with colloidal gold particles

The S protein was conjugated with 40 nm gold nanoparticles (nanoComposix, San Diego, CA, USA). Briefly, 45 µg of purified S protein in 10 mM of potassium phosphate (KP) buffer was incubated with a colloidal gold solution (pH 5.5) for 30 min, resulting in colloidal gold-labeled S, and then the mixture was centrifuged at 3,800 × g for 5 min at 4 °C to remove the supernatant. The pellet of colloidal gold-labeled S was resuspended by 125 µL of conjugate diluent solution. The optical density (OD) of the colloidal gold-labeled S was measured by spectrophotometry. The colloidal gold-labeled S was subsequently diluted to OD20 and stored at 4 °C until use.

To prevent false negative, a mouse IgG monoclonal antibody (mouse IgG mAb; Lampire Biological Laboratories, Pipersville, PA, USA) was used to bind goat anti-mouse IgG (Lampire Biological Laboratories, Pipersville, PA, USA) at control line. The mouse IgG mAb was then conjugated with colloidal gold solution as describe above, called colloidal gold-labeled mouse IgG mAb. After conjugation step, the colloidal gold-labeled mouse IgG mAb was diluted to OD10 and stored at 4 °C until use.

### 2.4. Assembly of the LFICS

Paper based LFICS was composed in sequence of backing card, sample pad, conjugate pad, analytical membrane and absorbent pad. All membranes were purchased from Whatman (Whatman International, UK). In the present study, the test strip was conducted separately for anti-PEDV IgA and IgG in order to evaluate antibodies against PEDV on each strip. Initially, G470 sample pad was overlapped with STD17 conjugate pad by 3 mm. Colloidal gold-labeled S at OD20 and colloidal gold-labeled mouse IgG mAb at OD10 were sprayed onto the conjugate pad.

CN95 nitrocellulose membrane analytical pad was sprayed 1 mg/mL of goat anti-mouse IgG (Lampire Biological Laboratories, Pipersville, PA, USA) served as control line (C-line) and 1 mg/mL of goat anti-pig IgA antibody and goat anti-pig IgG antibody (Abcam, Cambridge, UK) served as test line (T-line). This was followed by attaching the CF4 absorbent pad to the end of the test strip. All components were assembled on a 6 cm-length of polyvinylchloride (PVC) backing pad and cut into 4 mm-wide strips.

### 2.5. The principle and procedure of the LFICS

**Figure 1.**
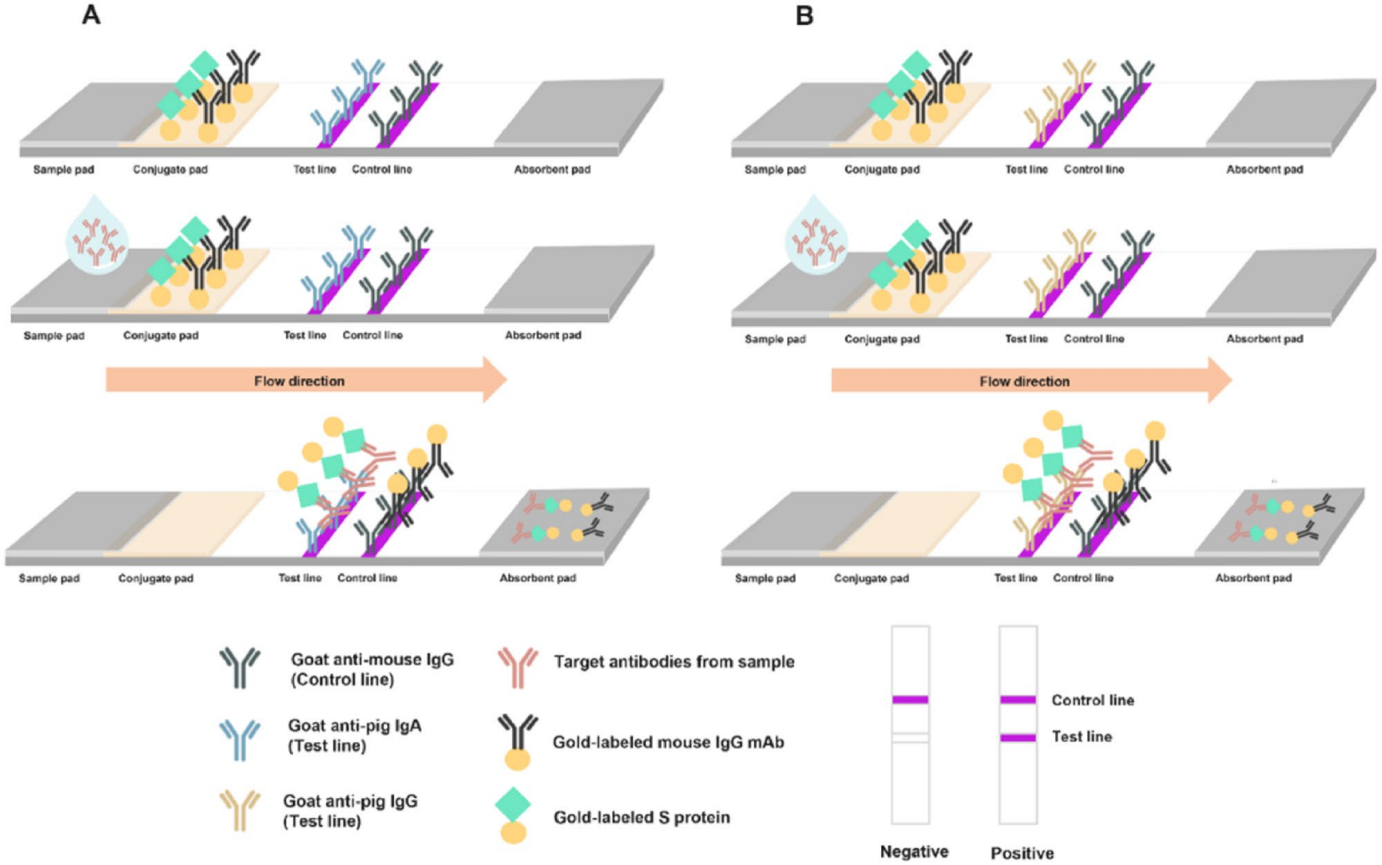
Schematic diagram of the LFICS. The LFICS consists of three pad components: sample pad, conjugate pad, and absorbent pad, along with a nitrocellulose membrane and a backing pad. Gold-labeled mouse IgG mAb and gold-labeled S protein are applied to the conjugate pad. The nitrocellulose membrane features two lines: test line and control line. The test line included goat anti-pig IgA antibody (A) and goat anti-pig IgG antibody (B), while the control line contained goat anti-mouse IgG antibody.

Colostrum and milk samples were diluted in a 1:5 ratio with TWS (25 mM Tris base, 1% sodium triphosphate contaning 2% Tween-20) buffer, in a total volume of 100 µL, and then mixed to ensure homogeneity. The diluted sample was processed onto the sample pad of the LFICS and allowed to diffuse laterally at room temperature for 15 min. Results were observed using naked eyes or a RapidScan ST5 Reader (Pacific Image Electronics, New Taipei, Taiwan). If the sample contained target antibodies (anti-PEDV specific IgA or IgG), these antibodies formed a complex with the gold-labeled S protein, which then bound to goat anti-pig IgA or IgG on the T-line, producing a red to magenta color. The C-line displayed color due to complex of gold-labeled mouse IgG mAb and goat anti-mouse IgG antibodies, indicating a positive result. If the sample did not contain target antibodies, only the C-line would show color, indicating negative result. The presence or absence of color at the C-line determined the validity of the LFICS; a visible C-line indicated a valid test, while its absence indicated an invalid test. A schematic diagram of the LFICS is shown in Figure 1.

### 2.6. Validation of limit of detection (LOD) of the LFICS

To evaluate the LOD of the LFICS, an anti-PEDV positive sample was serially diluted two-fold from 1:5, 1:10, 1:20, 1:40, 1:80 and 1:160, respectively. Each individual LFICS was then immersed in a separate diluted sample and incubated for 15 min. After the appearance of color on the C-line and T-line, the density of the lines on the LFICS was measured using the RapidScan ST5 Reader (Pacific Image Electronics, New Taipei, Taiwan).

To evaluate the LOD of VN level, an anti-PEDV positive sample was serially diluted ten-fold from 4log_2_, 3log_2_, 2log_2_, 1log_2_ and 0log_2_, respectively. The diluted samples were vertically diffused through a test strip and then incubated for 15 min. RapidScan ST5 Reader (Pacific Image Electronics, New Taipei, Taiwan) was used to measure intensity of C- and T-line.

For studying cross-reactivity, both anti-PEDV positive and anti-PDCoV positive samples, as well as anti-PEDV negative and anti-PDCoV negative samples, were utilized. These samples were diluted in TWS buffer at a ratio of 1:5 and tested using the LFICS. Subsequently, the density of the lines on the LFICS was measured using the RapidScan ST5 Reader (Pacific Image Electronics, New Taipei, Taiwan).

### 2.7. Semiquantitative (visual scores) and quantitative evaluation of antibody against PEDV

A visual score is a method of assessment based on visual observation of the colors developed on the pad. To evaluate both semiquantitative (visual scores) and quantitative aspects, 188 samples were diluted in a 1:5 ratio in TWS buffer and tested using the LFICS. After the LFICS was immersed in the diluted milk sample, visual results were scored semi-quantitatively as follows: no visibility of the T-line was assigned a score of 0, tests with a T-line weaker than the C-line were assigned a score of 1, tests with a T-line approximately equal in density to the C-line were scored 2, and tests with a T-line darker than the C-line were scored 3 (Chesnais et al. 2013).

A RapidScan ST5 Reader (Pacific Image Electronics, New Taipei, Taiwan) was used to measure the intensity of the C- and T-lines. The measurements were performed immediately after the visual reading, that is, 15 min after placement of the milk on the sample pad. The quantitative ratio was defined as ΔMargentaT-line divided by ΔMargentaC-line (T/C ratio).

### 2.8. Determination of performance of the LFICS on field samples

The LFICS was used to test 188 samples. All samples were evaluated for anti-S antibody levels against PEDV using an in-house indirect ELISA. The relative sensitivity and specificity of the LFICS compared to ELISA were calculated as the ratio of true positives to the total number of positive samples and true negatives to the total number of negative samples, respectively. Agreement between ELISA and the LFICS was analyzed using the Cohen’s kappa value.

### 2.9. Stability and repeatability test of the LFICS

The LFICS were tested for stability and repeatability using both anti-PEDV positive and negative samples, including IgA and IgG. The LFICS were stored at RT for 6 and 12 months to assess their stability. To evaluate repeatability, all tests were conducted in triplicate. Test results were interpreted by measuring the intensity of T-line and C-line with a RapidScan ST5 Reader (Pacific Image Electronics, New Taipei, Taiwan), and the T/C ratio was calculated. Coefficient of variation (CV) was determined for each storage time point as the ratio of the standard deviation (SD) to average value from the three independent tests. A CV of less than 15% indicated acceptable repeatability (Geng et al. 2022).

### 2.10. Samples

One hundred and eighty-eight samples, comprising 82 colostrum and 106 milk samples, were collected from swine farms located in the western and south regions of Thailand. Colostrum samples were collected immediately after farrowing (less than 24 h). Milk samples were collected from day 1 to day 14 after farrowing. All samples were stored at −20 °C until use. Neutralization (VN) assay is the gold standard for evaluating neutralizing antibodies in samples.

All samples were initially subjected to confirmation of positive and negative status by VN assay subsequently tested by indirect S-ELISA for detection of specific IgA and IgG against PEDV, as well as LFICS.

### 2.11. Cells, virus and viral neutralization (VN) assay

Vero C1008 cells were obtained from ATCC (ATCC^®^ CRL-1586^TM^). Vero C1008 cells were cultured in maintenance media (Dulbecco’s Modified Eagle Medium; DMEM supplemented with antibiotics: 100 units/mL of penicillin, 100 μg/mL of streptomycin, and 0.25 μg/mL of Fungizone^®^ (Life Technologies, New York, USA), and 10% heat inactivated fetal bovine serum (Gibco, New York, USA) in T75 flask (Corning^®^, New York, USA) and maintained at 37 °C in a humidified 5% CO_2_ incubator.

PEDV isolate SBPED0211_1 (GenBank: accession number KC764956) was used in this study (Srijangwad et al. 2021).

All samples were heat-inactivated at 56 °C for 30 min before performing the VN assay. VN assay was performed as previously described (Srijangwad et al. 2021). Briefly, samples were serially diluted in a 2-fold dilution from 1:2 to 1:256 in maintenance media, totaling 50 µL (DMEM supplemented with antibiotics: 100 units/mL of penicillin, 100 μg/mL of streptomycin, and 0.25 μg/mL of Fungizone^®^ (Life Technologies, New York, USA), and 8 µg/ml trypsin/EDTA (Gibco, New York, USA)). Fifty microliters of diluted PEDV was added into wells containing the diluted samples. The mixture of PEDV and diluted samples were incubated for 1 h at 37 °C with 5% CO_2_. When the Vero C1008 monolayer cells in a 96-well microplate (Corning^®^, New York, USA) reached 80% confluence, they were washed once with 1X PBS (1X phosphate-buffered saline; 0.1 M, pH 7.2) and once with maintenance media before adding the mixture of PEDV-diluted samples. The cells containing the mixture of PEDV-diluted samples were then incubated at 37 °C with 5% CO_2_ for 3 days. To calculate VN titer, the cells were fixed using cold acetone-methanol and then subjected to indirect immunofluorescence assay (IFA). The protocol and analysis were described as previously reported (Srijangwad et al. 2017). Samples with a VN titer ≥ 2log_2_ were determined to be VN positive, while those with a VN titer ˂ 1log_2_ were determined to be VN negative (Srijangwad et al. 2021).

### 2.12. Indirect enzyme-linked immunosorbent assay (ELISA)

The in-house indirect ELISA for detection of IgA and IgG based on the S protein was utilized to assess the performance of the LFICS. The indirect ELISA method was describes previously (Srijangwad et al. 2021). In brief, the S protein was diluted in bicarbonate buffer, pH 9.6, and then coated onto a 96-well immuno plate (Nunc, Thermo Fisher Scientific, Waltham, MA, USA), followed by overnight incubation at 4 °C. After removing the S protein, the plate was blocked with 5% (w/v) BSA (Merck, Darmstadt, Germany) in 0.05% PBST (1X PBS, pH 7.4 contaning 0.05% Tween-20) for 3 h at 37 °C. The samples were diluted to 1:200 in 0.05% PBST containing 10% (v/v) goat serum and added onto the plate, then was incubated for 1 h at 25 °C. Following a washing step, HRP-conjugated goat anti-pig IgA and IgG (1:10,000) (Abcam, Cambridge, UK) were added and incubated for 1 h at 25 °C. After washing off the secondary antibodies, 3,3′,5,5′- TMB substrate (Thermo Fisher Scientific, Waltham, MA, USA) was added and incubated in the dark for 5-15 min at 37 °C. The reaction was stopped by adding 50 µL of 1 normal H_2_SO_4_ to each well, and the OD were measured using an ELISA plate reader at 450 nm. (Metertech, Taipei, Taiwan). S/P ratio represented the immune response levels in the samples. Sample is considered positive for both IgA and IgG when the S/P ratio > 0.45, as determined by receiver operating characteristic (ROC) curve analysis.

### 2.13. Statistical analysis

The performance of the LFICS was compared with the results of VN and ELISA. Pearson’s correlation was used to evaluate the relationship between the VN assay, ELISA S/P ratio, and the LFICS in quantitative (T/C ratios). Differences in mean T/C ratios and visual scores were analyzed using Cuzick’s trend test. Agreement between ELISA and LFICS was assessed using Cohen’s kappa coefficient (κ). All statistical analyses were performed using GraphPad Prism 10 (GraphPad Software Inc., La Jolla, CA). A *P* value ≤ 0.05 was considered statistically significant.

## 3. Results

### 3.1. Confirmation of recombinant S protein by Native-PAGE and immunoblotting

**Figure 2.**
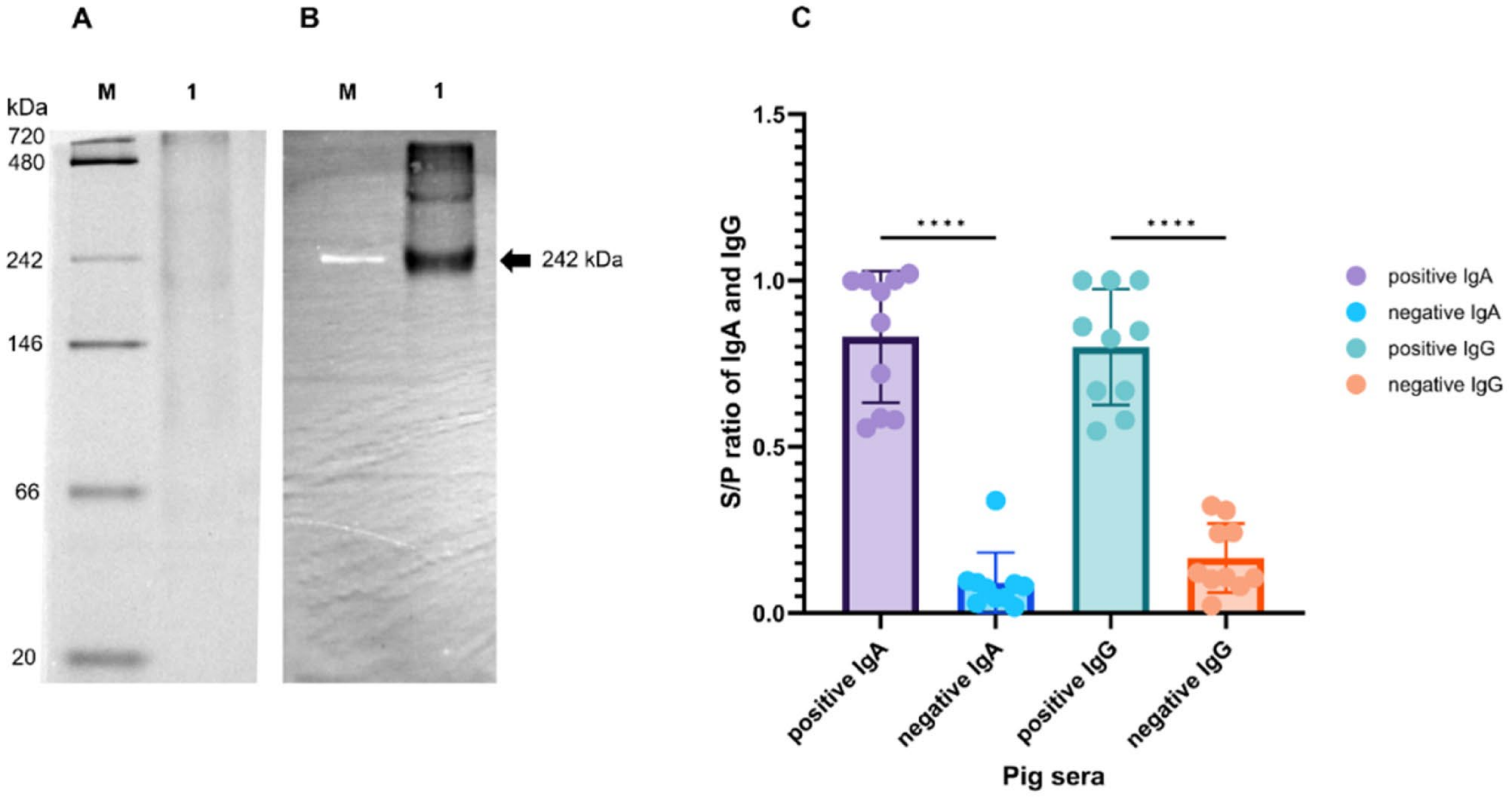
Expression and purification of recombination S protein. (A) Purification profile of S protein. Lane M: NativeMARK^™^ Unstained Protein Standard molecular weight marker (20-720 kDa); lane 1: purified SP protein. (B) Analysis of purified S protein by immunoblotting. Lane M: NativeMARK^™^ Unstained Protein Standard; lane 1: anti-6xHis antibody. (C) Antigenic reactivity of S protein to positive and negative sera against PEDV was evaluated using an indirect ELISA, measured by S/P ratio.

To determine S protein expression, a suspension culture of Sf9 cells was infected with recombinant-S baculovirus carrying 6XHis-tagged fusion at MOI of 2. The supernatant was purified using affinity chromatography on a HisTrap^™^ HP column. The 6XHis-tagged S protein was eluted from the Ni sepharose resin with 100 mM imidazole, followed by desalting using an Amicon Ultra-15 centrifugal filter with a 50 kDa cutoff. The purified S protein was then subjected to Native-PAGE and immunoblotting to confirm protein expression (Figure 2A,B). The results demonstrated a well-defined band of high purity for the S protein, approximately 242 kDa, as displayed on an 8% polyacrylamide gel (Figure 2A) and a PVDF membrane (Figure 2B).

To analyze biological antigenic reactivity of the S protein, we used pig sera against PEDV to determine S protein reactivity through indirect ELISA (Figure 2C). The ELISA results revealed that the S protein could significantly react with positive sera for both IgA and IgG against PEDV when compared to negative sera. This indicates that the recombinant S protein can recognize antibodies against PEDV and is suitable for the development of diagnostic devices.

### 3.2. Limit of detection and cross-reactivity of the LFICS

**Figure 3.**
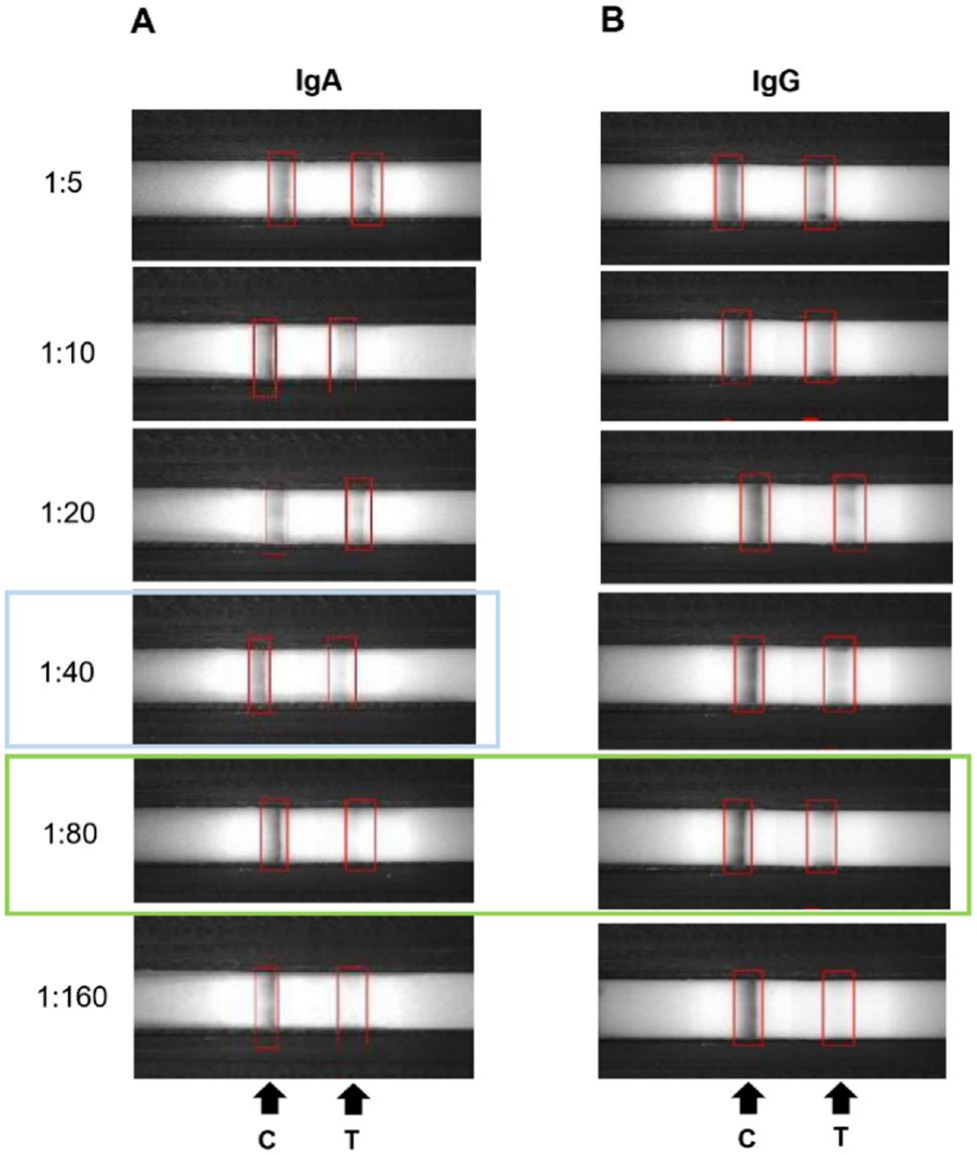
Limited of detection (LOD) of the immunochromatographic strip test. Different dilution of anti-PEDV specific IgA and IgG positive milk (1:5, 1:10, 1:20, 1:40, 1:80 and 1:160). The strip detection limits were observed to be 1:40 for IgA (A) and 1:80 for IgG (B), as indicated by the blue and green boxes, respectively.

Limit of detection (LOD) of the LFICS is shown in Figure 3. Anti-PEDV positive sample was two-fold serially diluted to examine the sensitivity. The LFICS had a sensitivity up to 1:40 and 1:80 for specific IgA and IgG, respectively.

**Figure 4.**
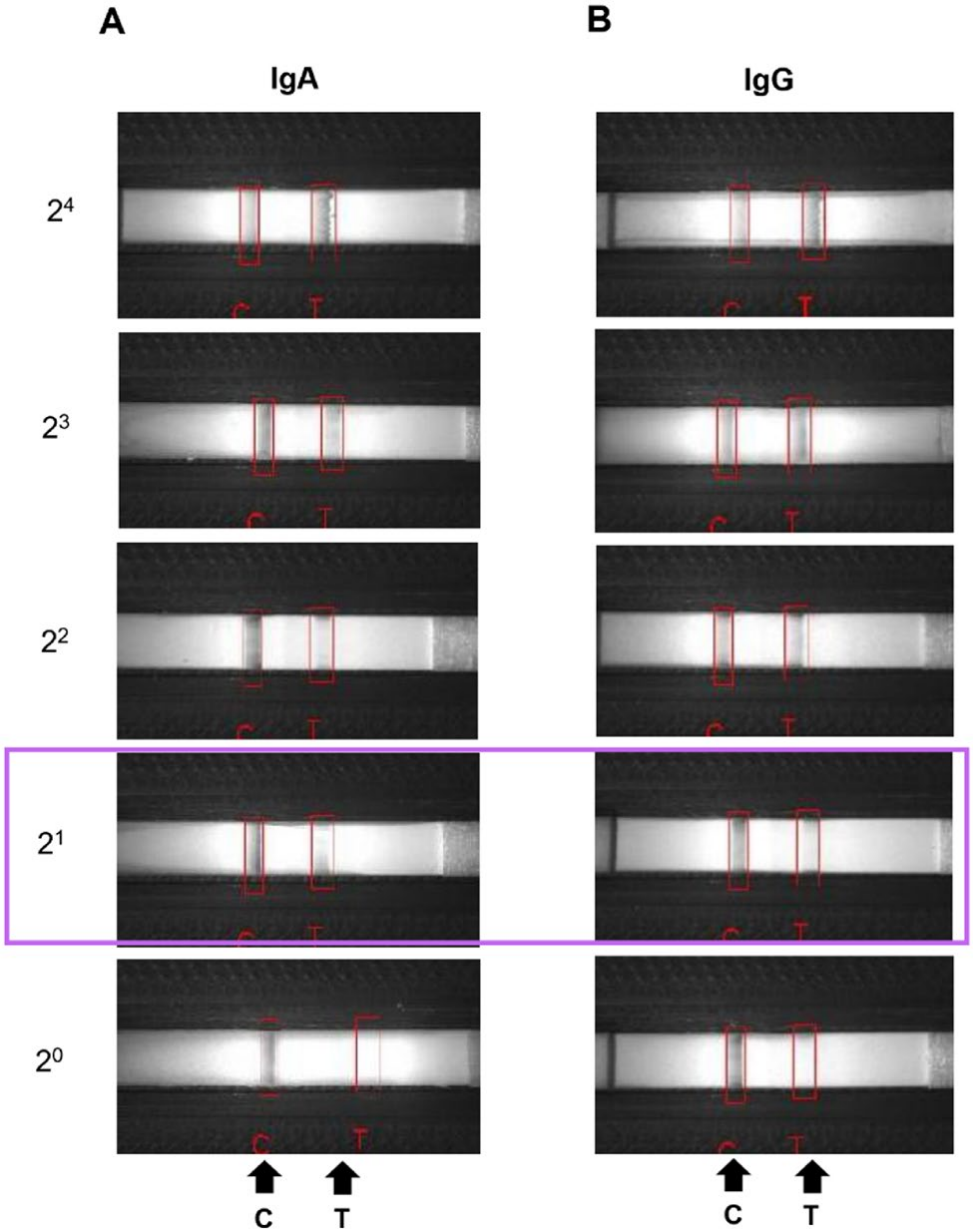
Limited of detection (LOD) of neutralizing antibody (nAb) levels against PEDV on immunochromatographic strip test. Positive nAb sample was diluted ten-fold at 4log_2_, 3log_2_, 2log_2_, 1log_2_ and 0log_2_, respectively. The sensitivity of the specific IgA (A) and IgG (B) strips was 1log_2_ (purple box).

Known levels of neutralizing antibodies (nAbs) were obtained from VN assay. Positive milk sample containing anti-PEDV nAbs was diluted ten-fold to test LOD using LFICS. The LOD of nAb levels is shown in Figure 4. Both the specific IgA and IgG strips of the LFICS had LOD at 1log_2_. The results indicate that the LFICS had high LOD in detecting nAb levels.

**Figure 5.**
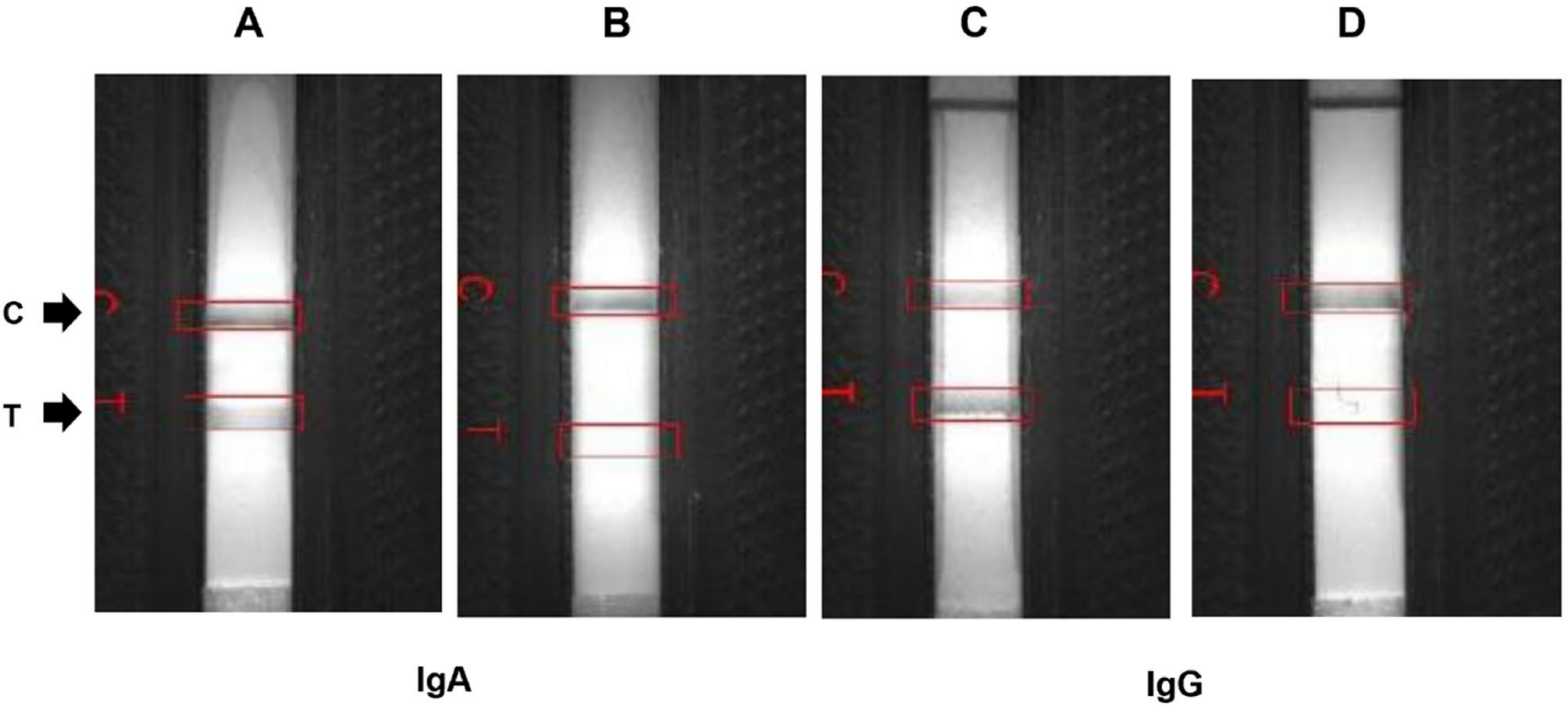
Cross-reactivity of the immunochromatographic strip test. The strip detection of anti-PEDV specific IgA (A) and IgG (C) positive milk, as well as anti-PDCoV specific IgA (B) and IgG (D) positive milk. The samples were diluted at 1:5 in TWS buffer, and then each strip was immersed with the dilution sample. Positive signals were observed on T-line of the anti-PEDV specific IgA (A) and IgG (C) positive milk samples. C and T represent control line and test line, respectively.

Cross-reactivity of the LFICS is shown in Figure 5. The anti-PEDV specific IgA positive sample, anti-PEDV specific IgG positive sample, anti-PDCoV specific IgA positive sample, anti-PDCoV specific IgG positive sample and anti-PEDV negative sample were used to determine cross-reactivity of the LFICS. The anti-PEDV positive sample showed positive band at the T-line both specific IgA and IgG test strips. The anti-PDCoV positive samples and anti-PEDV negative sample were negative at T-line.

### 3.3. Correlation between VN assay, ELISA and the LFICS

**Figure 6.**
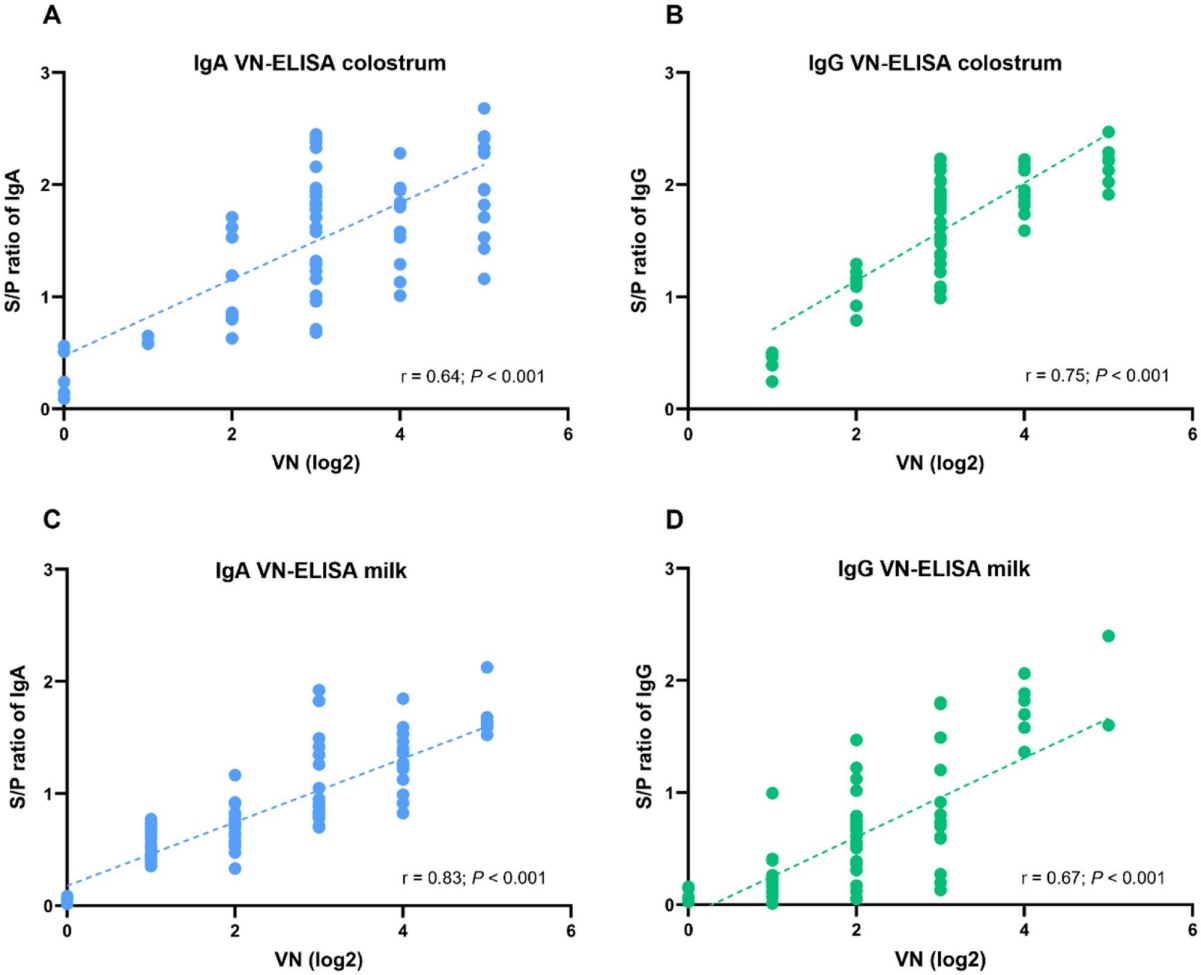
Correlation of antibody levels between VN titer and ELISA S/P ratio. A scatter plot of antibody levels in colostrum (A,B) and milk (C,D) is shown. Linear regression, r and P values were analyzed by Pearson (two-tails) with a fitted regression line and 95% confidence intervals.

All 188 samples were evaluated for antibody levels against PEDV using VN, ELISA, and the LFICS. The results of antibody levels from ELISA were compared with titer of VN as a gold standard to estimate protection levels in colostrum and milk samples. Correlations between VN and ELISA were illustrated in Figure 6, with results indicated by regression lines and correlation coefficients (r). The correlation coefficients between VN and ELISA of specific IgA in colostrum and milk were 0.64 (Figure 6A) and 0.83 (Figure 6C), respectively. In Figure 6B,D, the correlation coefficient between the two assays of specific IgG in colostrum and milk were 0.75 and 0.67, respectively.

**Figure 7.**
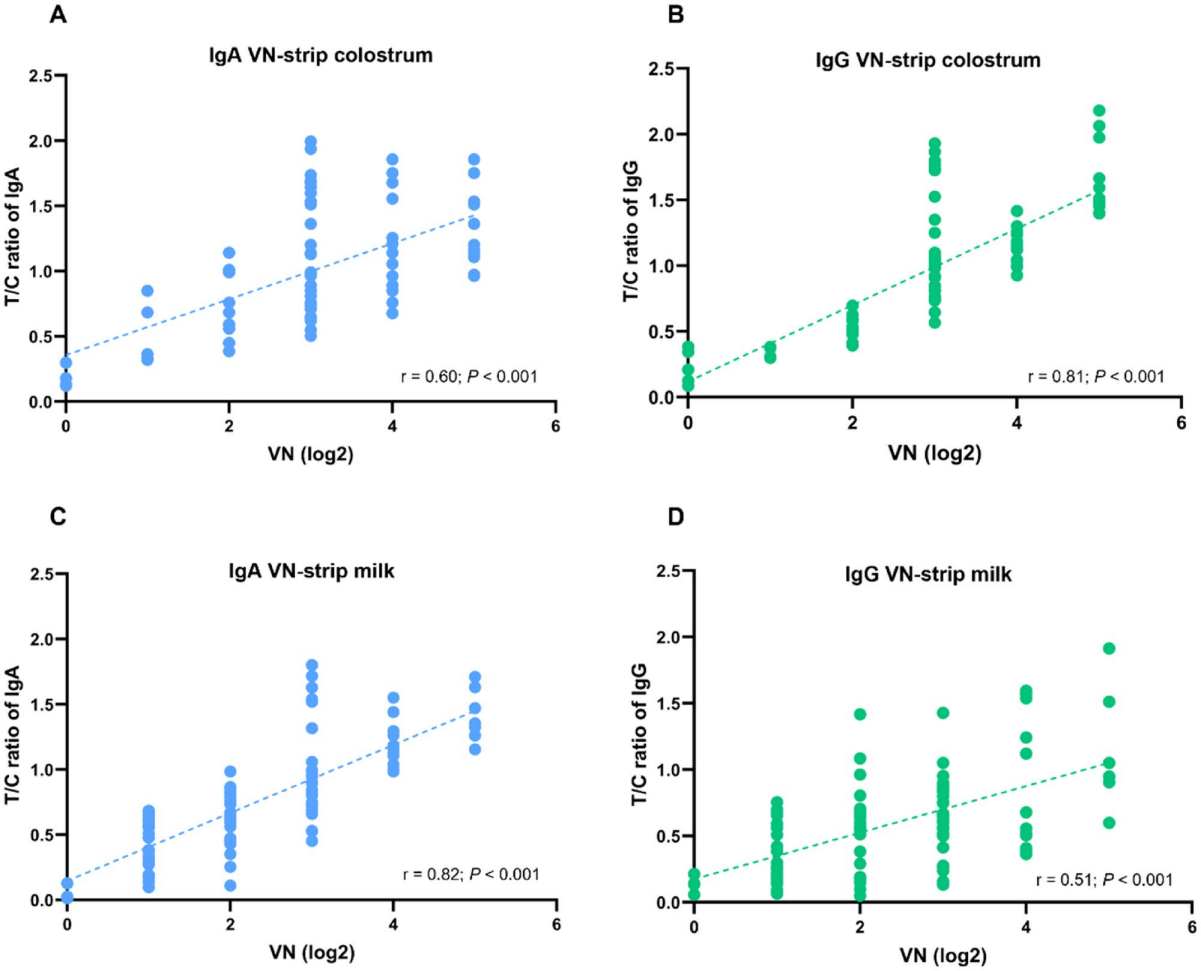
Correlation of antibody levels between VN titer and strip test T/C ratio. A scatter plot of antibody levels in colostrum (A,B) and milk (C,D) is shown. Linear regression, r and P values were analyzed by Pearson (two-tails) with a fitted regression line and 95% confidence intervals.

Levels of antibodies measured by the LFICS were compared to values obtained from VN assay. Figure 7 illustrated the correlation between VN titers and T/C ratios of the LFICS. For specific IgA, the correlation coefficients were 0.60 in colostrum (Figure 7A) and 0.82 in milk (Figure 7C). For specific IgG, the correlation coefficients were 0.81 in colostrum (Figure 7B) and 0.51 in milk (Figure 7D).

**Figure 8.**
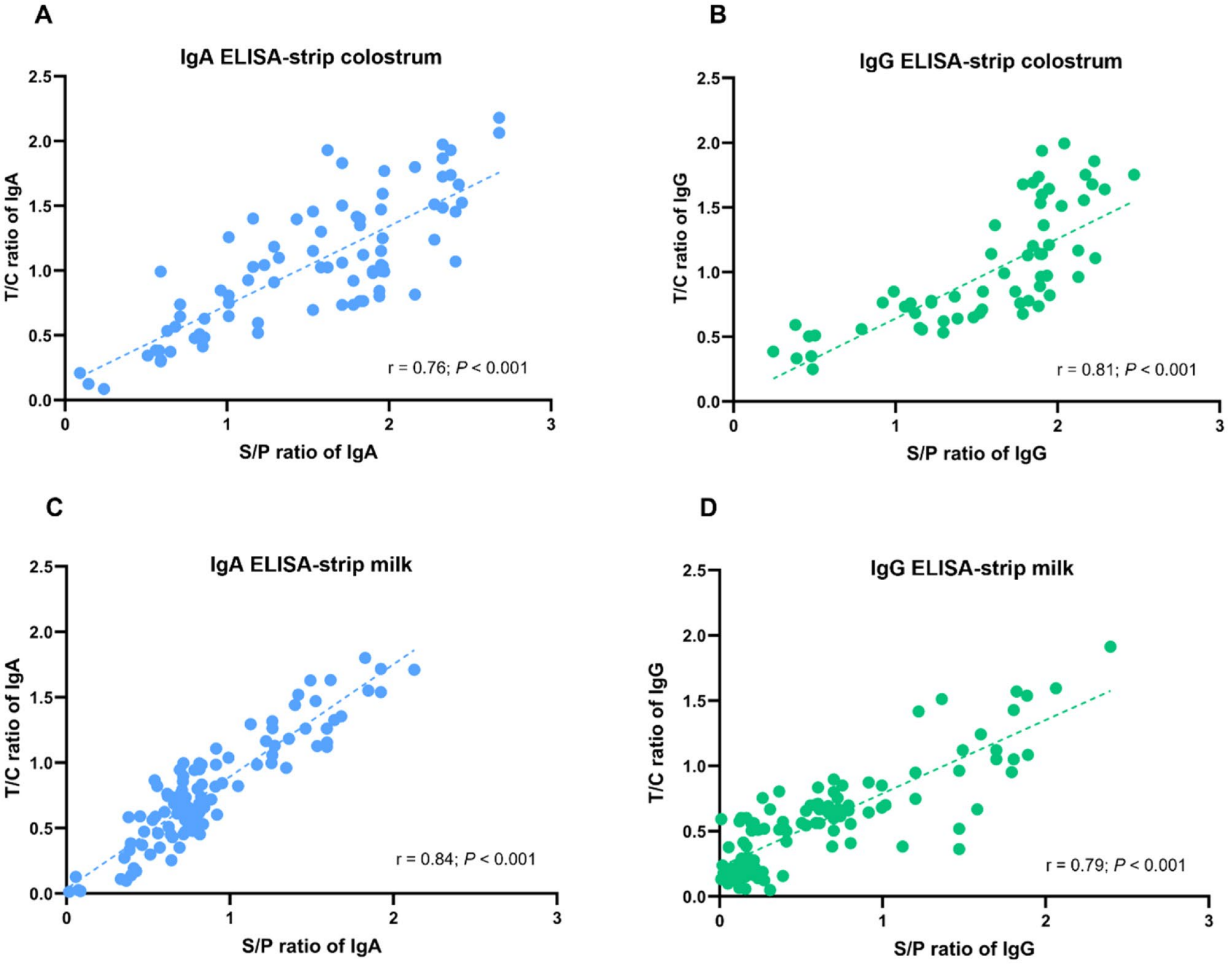
Correlation between ELISA S/P ratio and strip test T/C ratio. Levels of IgA (A,C) and IgG (B,D) antibodies against PEDV in pig colostrum and milk were evaluated using ELISA and strip test. All samples were analyzed with Pearson r and P values (two-tails) with a fitted regression line and 95% confidence intervals.

Correlation between ELISA and the LFICS was displayed in Figure 8. The correlation coefficients of specific IgA values were 0.76 in colostrum (Figure 8A) and 0.84 in milk (Figure 8C). For IgG values, correlation coefficients were 0.81 in colostrum (Figure 8B) and 0.79 in milk (Figure 8D).

The results indicated that the LFICS exhibited a high correlation with VN and ELISA, suggesting that the LFICS could be used instead of both these standard methods in field conditions.

### 3.4. Relationship between semiquantitative (visual scores) obtained from the LFICS and quantitative levels measured by ELISA (S/P ratio) and the LFICS (T/C ratio)

**Figure 9.**
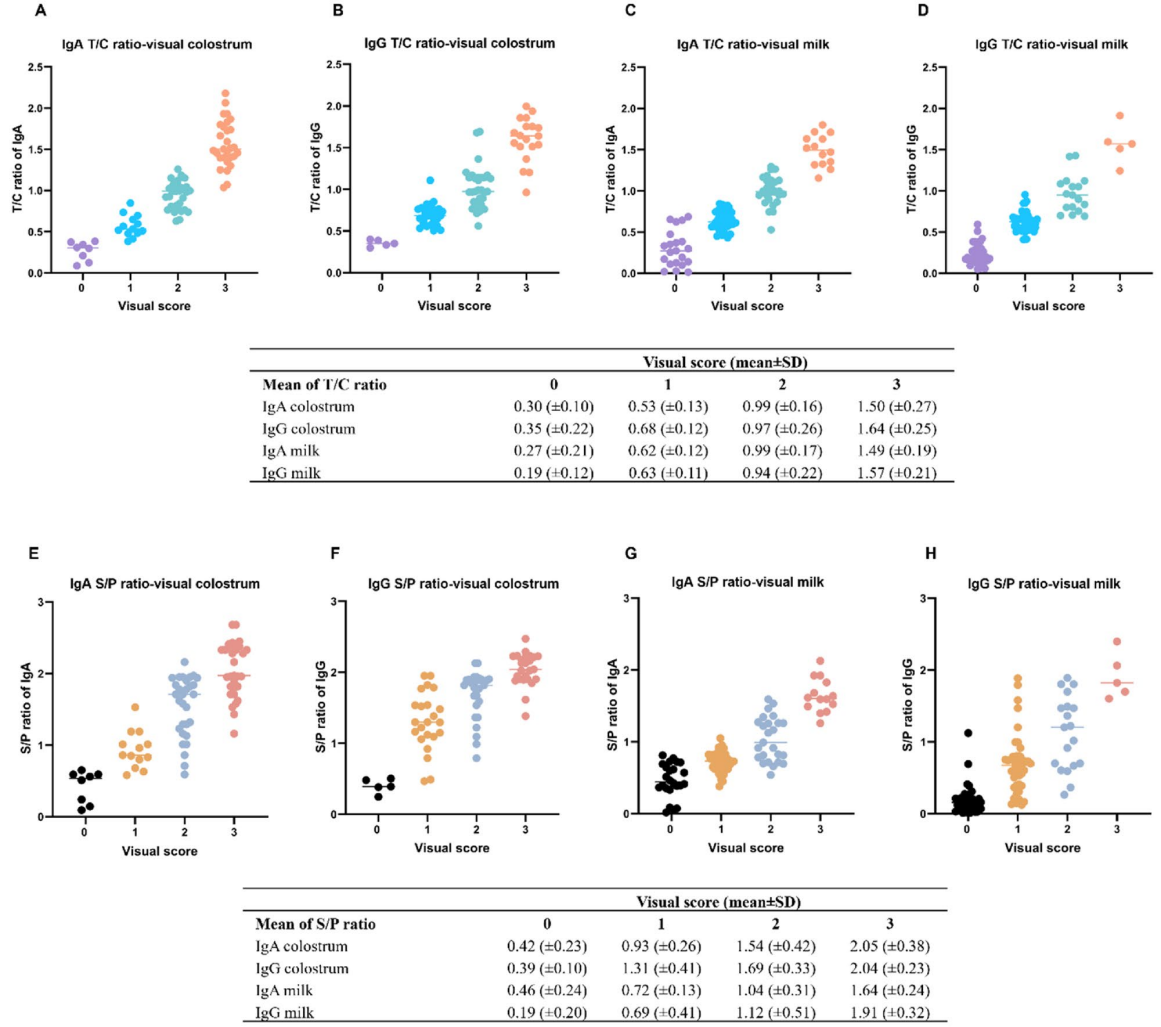
Relationship between semiquantitative (visual scores) and quantitative measurements by ELISA and test strip. Relationship between visual scores and T/C ratio by the test strip for IgA and IgG ELISA in colostrum (a and B) and milk (C and D), respectively. Relationship between visual scores and S/P ratio by ELISA for IgA and IgG ELISA in colostrum (E and F) and milk (G and H), respectively. Horizontal lines represent as mean ± SD using Cuzick’s trend test with 95% confidence interval.

Relationship between semiquantitative (visual scores) and quantitative assessment results of the ELISA and the LFICS are presented in Figure 9. Antibody levels by the LFICS and ELISA were compared with visual scores of 0, 1, 2, and 3, showing mean (± standard deviation; SD) using Cuzick’s trend test (*p* < 0.001).

The T/C ratio of the individual LFICS was compared with visual scores of 0, 1, 2, and 3, showing mean T/C ratios (±SD). Colostrum samples had mean T/C ratios of specific IgA: 0.30 (±0.10), 0.53 (±0.13), 0.99 (±0.16), and 1.50 (±0.27) (Figure 9A). Mean T/C ratios of specific IgG were 0.35 (±0.22), 0.68 (±0.12), 0.97 (±0.26), and 1.64 (±0.25) (Figure 9B) corresponding to visual scores of 0, 1, 2, and 3, respectively. Milk samples from individuals with visual scores of 0, 1, 2, and 3 had mean T/C ratios of 0.27 (±0.21), 0.62 (±0.12), 0.99 (±0.17) and 1.49 (±0.19) for specific IgA (Figure 9C) and 0.19 (±0.12), 0.63 (±0.11), 0.94 (±0.22) and 1.57 (±0.21) for specific IgG, respectively (Figure 9D).

To compare visual scores with ELISA as reference method. Visual score of 0, 1, 2, and 3 obtained from the LFICS were compared with mean S/P ratios (±SD) from ELISA. In colostrum, IgA values were 0.42 (±0.23), 0.93 (±0.26), 1.54 (±0.42), and 2.05 (±0.38) (Figure 9E), and IgG values were 0.39 (±0.10), 1.31 (±0.41), 1.69 (±0.33), and 2.04 (±0.23) (Figure 9F). The mean (±SD) S/P ratios of milk samples were 0.46 (±0.24), 0.72 (±0.13), 1.04 (±0.31), and 1.64 (±0.24) for IgA (Figure 9G). S/P ratios for IgG were 0.19 (±0.20), 0.69 (±0.41), 1.12 (±0.51), and 1.91 (±0.32) (Figure 9H).

### 3.5. Relative sensitivity and relative specificity of the LFICS compared with ELISA

All colostrum and milk samples were evaluated for antibody levels specific to IgA and IgG using ELISA and the LFICS. The antibody levels measured by the LFICS were compared with those obtained by ELISA, which served as the reference method, to calculate the relative sensitivity and specificity of the LFICS in both sample types (Table 1.).

**Table 1.** Relative sensitivity and relative specificity of the LFICS compared with ELISA.

			ELISA			
Sample type	Isotype	LFICS	Positive	Negative	Total	Kappa value
Colostrum	IgA	Positive	78^a^	1	79	0.654
		Negative	1	2^c^	3	
		Total	79^b^	3^d^	82	
		% Relative sensitivity	98.73			
		% Relative specificity	66.67			
					
	IgG	Positive	75^a^	1	78	0.737
		Negative	1	3^c^	4	
		Total	78^b^	4^d^	82	
		% Relative sensitivity	96.15			
		% Relative specificity	75.00			
Milk	IgA	Positive	87^a^	3	90	0.730
		Negative	4	12^c^	16	
		Total	91^b^	15^d^	106	
		% Relative sensitivity	95.60			
		% Relative specificity	80.00			
					
	IgG	Positive	73^a^	3	76	0.586
		Negative	13	17^c^	30	
		Total	86^b^	20^d^	106	
		% Relative sensitivity	84.88			
		% Relative specificity	85.00			

^a^The number of true positives.

^b^The total number of positive samples.

^c^The number of true negatives.

^d^The total number of negative samples.

%Relative sensitivity = (a/b)*100; % Relative specificity = (c/d)*100.

In colostrum samples, the LFICS showed a relative sensitivity of 98.73% (78/79) and a specificity of 66.67% (2/3) for specific IgA detection. For IgG detection, the LFICS exhibited a relative sensitivity of 96.15% (75/78) and a specificity of 75.00% (3/4).

In milk samples, the percentage of relative sensitivity and specificity of the LFICS were 95.60% (87/91) and 80.00% (12/15) for IgA, respectively, while IgG were 84.88% (73/86) and 85.00% (17/20) for relative sensitivity and specificity of the LFICS, respectively.

Comparing ELISA and the LFICS using kappa analysis, moderate agreement was observed in colostrum for IgA (κ = 0.654) and in milk for IgG (κ = 0.586). Substantial agreement with ELISA method was found in colostrum for IgG (κ = 0.737) and in milk for IgA (κ = 0.730).

### 3.6. Stability and repeatability of the LFICS

**Figure 10.**
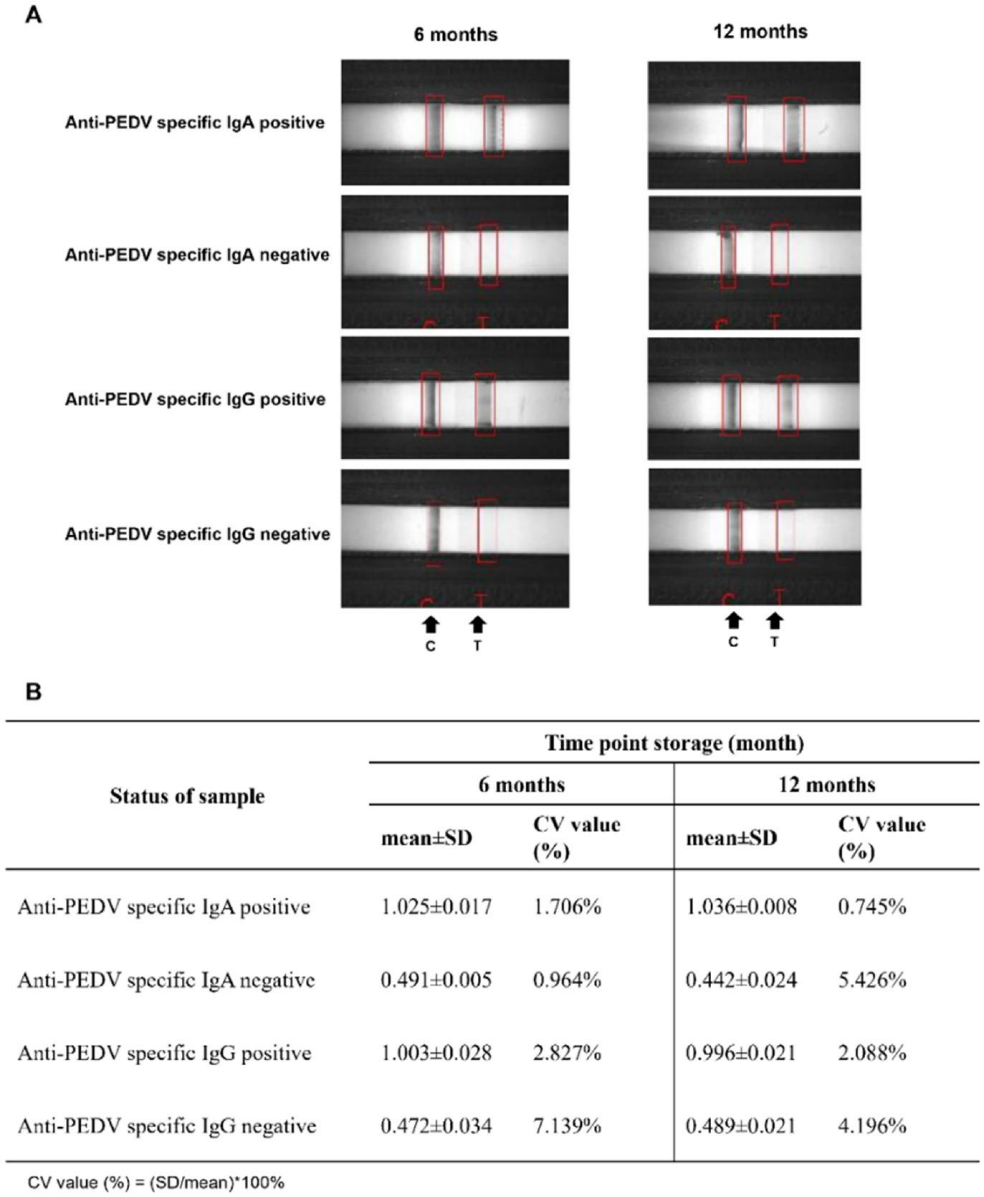
Stability and repeatability tests of the LFICS. The LFICS demonstrated stability for 12 months at RT (A). Repeatability was determined using CV percentage at different storage times (B).

The LFICS were stored at room temperature for 6 and 12 months to evaluate their stability (Figure 10A). The results showed that the LFICS were stable for 12 months at RT for both specific IgA and IgG LFICS, demonstrating that the LFICS device is practical and suitable for long-term storage. Additionally, repeatability was tested for all LFICS at each time point in triplicate (Figure 10B). All CV values were less than 15%, indicating that the LFICS has good repeatability in measuring antibody levels against PEDV.

## 4. Discussions

An important step in controlling PEDV damage in piglets is monitoring the lactogenic immunity response in sows’ colostrum and milk. It is crucial not only to understand how to prevent PEDV but also to be familiar with its history in herds and farms.

Viral neutralizing (VN) assay is an *in vitro* method used to detect neutralizing antibodies (nAbs) that function to neutralize viruses and prevent infection, indicating potential protective immunity (Vincent et al. 2006). Therefore, measuring levels of nAbs in colostrum and milk can provide insights into protective immunity. Generally known that the VN assay detects total antibody response in samples but does not differentiate between types of antibodies present, which may include IgG, IgA, and IgM, in colostrum and milk (Hurley and Theil 2011). To further analyze immune response following vaccination or post-infection, it is important to evaluate specific types of immunoglobulins (Ig) present. Methodologies such as ELISA can be employed to determine the types of antibodies in a sample. However, both methods require sophisticated laboratory equipment, biosafty considerations and time consuming.

To facilitate rapid management of PEDV on farms, a new detection tool was developed to replace traditional methods such as VN and ELISA. The lateral flow immunochromatographic strip (LFICS), which is fundamentally similar to ELISA, is a paper-based device that provides rapid, real-time, on-site detection and is user-friendly (Murray and Mace 2020). In the present study, we successfully developed a LFICS to evaluate levels of IgA and IgG in colostrum and milk based on S protein. The results of this LFICS showed a positive correlation with both the VN assay and indirect ELISA. The LFICS demonstrated a high limit of detection (LOD) for specific IgA and IgG, with no cross-reactivity with PDCoV. Moderate to substantial agreement, as revealed by kappa analysis, was observed compared with ELISA values. Additionally, semiquantitative visual scores were used to quantify antibody levels in colostrum and milk, and these scores correlated well with ELISA values. The individual scores indicated the mean antibody levels that provide protective immunity against PEDV. Our LFICS demonstrated a rapid and simple device for evaluating antibodies in field situations without the need for additional equipment.

Spike (S) protein is commonly used as an immunogen in vaccine development and antibody detection tools (Saif 1999; Chattha et al. 2015). The S protein-induced neutralizing antibodies found in colostrum and milk when administered intramuscularly as a vaccine and primed with attenuated TGEV orally (Park et al. 1998). In a previous study, the S1, M, E, and N structural proteins of PEDV were expressed for the detection of IgA and IgG antibodies in colostrum against PEDV, compared with VN assay (Song et al. 2023). They suggested that the S1 protein had a high correlation with VN titer followed by N, E and M proteins, respectively. Moreover, previous study reported that N protein does not contain neutralizing epitope, which subsequently resulted in more false-negative compared to the S protein in ELISA technique (Srijangwad et al. 2021). Thus, the LFICS using S protein conjugated with colloidal gold in this study can detect neutralizing antibodies against PEDV in colostrum and milk, facilitating accurate correlation of antibody titers compared to VN and ELISA methods, also providing faster results than those standard techniques (VN and ELISA). Additionally, the S protein used as an antigen in this study was produced from the PEDV G2 genotype. It shares a percentage identity of amino acids (aa) with the PEDV strain CV777 of 92.80%, while it is 8.30% for the PDCoV strain NT1_1215 (accession number KX361345). Based on these results, it is indicated that the LFICS can evaluate antibodies in both G1 and G2 genotypes of PEDV and does not exhibit cross-reactivity with PDCoV.

In this study, we identified different dominant Ig isotypes in colostrum and milk samples. Pearson’s correlation analysis revealed a stronger correlation for IgG compared to IgA in colostrum samples in VN-ELISA, VN- LFICS and ELISA- LFICS analyses. Conversely, IgA levels showed a higher correlation than IgG in milk samples. These findings suggest that IgG is abundant in the colostrum of sows, while IgA is abundant in their milk. This was supported by a previous study where colostrum was collected at 1, 6, 12, and 24 h, and milk was collected at 6, 12, 18, and 28 days after parturition. Total IgA and IgG were measured using commercial ELISA kit. The results showed that all Ig levels were highest at 1 h, with IgG dominating in colostrum at 98.17 mg/mL. Throughout the lactation period, the concentration of all Ig types decreased. Specifically, IgG levels decreased 27 times from the concentration observed in colostrum at 1 h, while IgA increased instead of IgG and persisted until the end of lactation (Markowska-Daniel et al. 2010). Additionally, the abundance of Ig isotypes depends on the method of stimulation (Srijangwad et al. 2017).

Two other qualitative rapid LFICS for detection of antibodies against PEDV have been reported. In a previous publication, they developed a LFICS to detect IgG in pig serum using N protein conjugated with colloidal gold particles (Li et al. 2018). However, the presence of antibodies in serum did not directly correlate with protection of piglet (Huang et al. 2013). Another study developed a LFICS for detecting secretory IgA (SIgA) in colostrum, using monoclonal anti-SIgA protein labeled with colloidal gold (Liu et al. 2020). However, the two studies mentioned were qualitative and did not assess semiquantitative visual scores for the easy evaluation of anti-PEDV levels under field conditions. The present study is the first report of relative visual scores compared with T/C ratio and ELISA values to evaluate antibody levels against PEDV in colostrum and milk for both IgA and IgG. The results demonstrated the mean (±SD) T/C ratio from the LFICS, which compares visual scores with S/P ratio ELISA values and could estimate antibody levels in colostrum and milk samples without the need for a reader machine. Therefore, the development of LFICS for evaluating IgA and IgG antibody levels in colostrum and milk provides a rapid, simple, sensitive, and specific tool for measurement passive immunity against PEDV infection and vaccination.

## Data Availability

Raw data were generated at Chulalongkorn University. Derived data supporting the findings of this study are available from the corresponding author on request.
